# Sagittal-spinopelvic alignment improves in patients with bilateral highly dislocated hip (Crowe type IV) after subtrochanteric shortening total hip arthroplasty: A retrospective radiographic study

**DOI:** 10.1097/MD.0000000000036966

**Published:** 2024-01-19

**Authors:** Tadatsugu Morimoto, Takaomi Kobayashi, Masatsugu Tsukamoto, Tomohito Yoshihara, Hirohito Hirata, Yu Toda, Masaaki Mawatari

**Affiliations:** a Department of Orthopaedic Surgery, Faculty of Medicine, Saga University, Saga, Japan.

**Keywords:** highly dislocated hip, hip-spine syndrome, hyperlordosis, sagittal spinopelvic alignment, total hip arthroplasty, total hip arthroplasty with subtrochanteric shortening osteotomy

## Abstract

In patients with bilateral highly dislocated hips (HDHs), total hip arthroplasty with subtrochanteric shortening osteotomy (S-THA) is a viable option for achieving adequate reconstruction with restoration of the anatomical hip center. This procedure has the potential to improve sagittal spinopelvic alignment (SSPA). However, reports are scarce owing to the rarity of this disease. The objective of this study is to investigate pre- and post-operative SSPA in patients with HDHs who had undergone S-THA. This retrospective radiographic study included 55 patients (54 females and 1 male; average age, 63.1 ± 6.9 years) who underwent S-THA. Lateral spine radiographs in the standing position were obtained pre- and post-operatively. The SSPA included lumbar lordosis (LL), sacral slope (SS), pelvic incidence (PI), and intervertebral disc (ID) angle of L1/2–L5/S. The SSPA pre- and post-S-THA was compared using a paired *t* test. Pearson correlation coefficient was used to assess the relationships between parameters. The mean pre- and post-operative LL and SS values were 62° and 49° (LL) and 50° and 39° (SS), respectively (*P* < .001). The ID angle was significantly reduced post-operatively at all levels (*P* < .001). The correlation coefficients between preoperative LL and SS and postoperative LL and PI were 0.81 and 0.38, respectively (*P* < .01). The preoperative SSPA of Crowe type IV HDHs revealed excessive pelvic anteversion and lumbar hyperlordosis, with a high correlation between LL and SS, suggesting that these alterations were compensatory changes to maintain body balance. Furthermore, in patients with HDHs and residual spinal flexibility, restoring the original pelvic morphology with S-THA may contribute to improved SSPA.

## 1. Introduction

**Figure 1.**
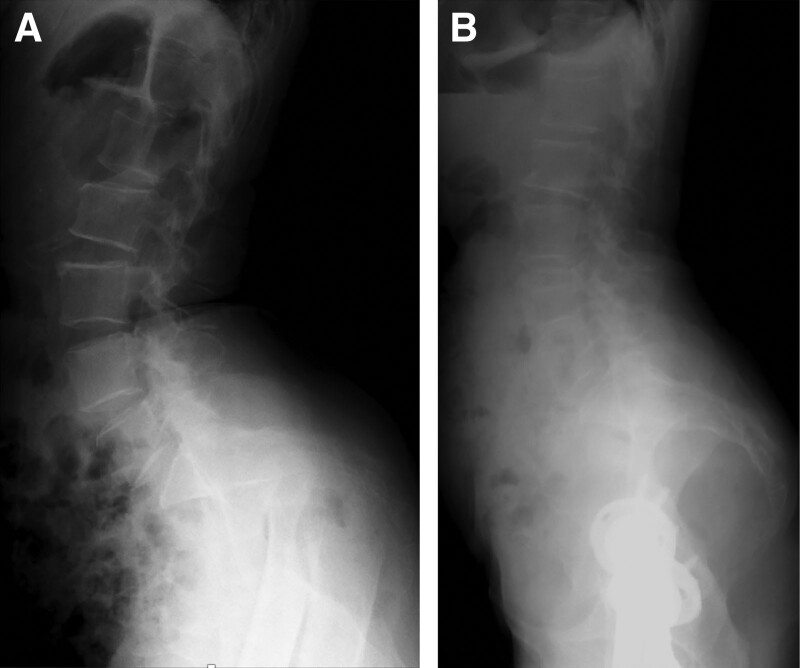
Decreased pelvic tilt and lumbar lordosis pre- and post-S-THA for HDHs. (A) Highly dislocated hip preoperative S-THA, LL(L1-S1) (84°) and SS (65°). (B) Highly dislocated hip post-operative S-THA. In patients with HDHs, after S-THA, which can restore the hip axis, a decrease in LL (L1-S1) (69°), SS (55°), and pelvic incidence (48°) can be detected. HDH, highly dislocated hip; LL, lumbar lordosis; SS, sacral slope; S-THA, subtrochanteric shortening total hip arthroplasty.

Hip disorders can affect spinal alignment, a phenomenon originally defined by Offierski and MacNab as “hip-spine syndrome.”^[1]^ However, the relationship between the hip and spine in this syndrome, regarding which structure is primarily involved, similar to the chicken-first or egg-first analogy, remains unclear. Therefore, investigation of spinal alignment in specific hip disease cases, such as ankylosed hips or highly dislocated hips (HDHs) (Fig. 1A and B), can help clarify the effect of hip lesions on spinal lesions, that is, the causation.^[2,3]^ A posterosuperior shift of the femoral head in the hips of patients with a Crowe type IV (>100% subluxation) HDH^[4]^ is compensated for by the anterior angulation of the pelvis and lumbar hyperlordosis.^[5]^ In patients with HDHs, total hip arthroplasty with subtrochanteric shortening osteotomy (S-THA) can help achieve adequate reconstruction with restoration of the anatomical hip center. This procedure has the potential to improve sagittal spinopelvic alignment (SSPA). However, reports are scarce owing to the rarity of this disease.^[6–8]^

The SSPA analysis involves measurement of the lumbar lordosis (LL: L1-S1), sacral slope (SS), pelvic tilt, and pelvic incidence (PI) as routine spinopelvic sagittal parameters.^[2]^ PI is defined as the angle between a line perpendicular to the midpoint of the sacral plate and the line connecting that point with the biaxial femoral head (hip axis).^[9]^ Since the measurement points of PI are composed of the spine and hip joints, and because they are invariant throughout life and unaffected by posture, PI is the most important indicator of the relationship between the spine and hip joints.^[8,9]^ However, accurate identification of the PI in patients with HDHs is impossible because of the deformity and dislocation of the femoral head (Fig. 1A).^[6,10]^

Although the PI can be measured after S-THA with an HDH, these measurements may be compromised due to difficulties in placing the THA cup in true acetabulum, difficulties in measuring the hip axis from the THA ball, and soft tissue imbalance (Fig. 1B).^[10,11]^ Takahashi et al^[10]^ reported that a new parameter-based PI prediction formula using the superior border of the pubic symphysis without the hip axis could predict the PI with high accuracy.

Investigations using this formula will help obtain the original PI in patients with HDHs, which has been previously unknown, and will contribute to a deeper understanding of the relationship between the lumbar spine and pelvis in these patients, thereby providing new knowledge of hip-spine syndrome. The primary objective of this hypothesis finding study was to investigate the SSPA and lumbar segmental anatomy in patients with HDH pre- and post-S-THA to determine if S-THA improves excessive pelvic anteversion and lumbar hyperlordosis in patients with HDH by restoring the original pelvic morphology.

## 2. Patients and methods

The present study was a single-center, retrospective, radiographic study. It was approved by the Institutional Review Board of our institution (#2023-04-*R*-08). Moreover, it adhered to the principles of the Declaration of Helsinki. Informed consent was obtained through the use of an opt-out form on the website.

### 2.1. Study participants

**Figure 2.**
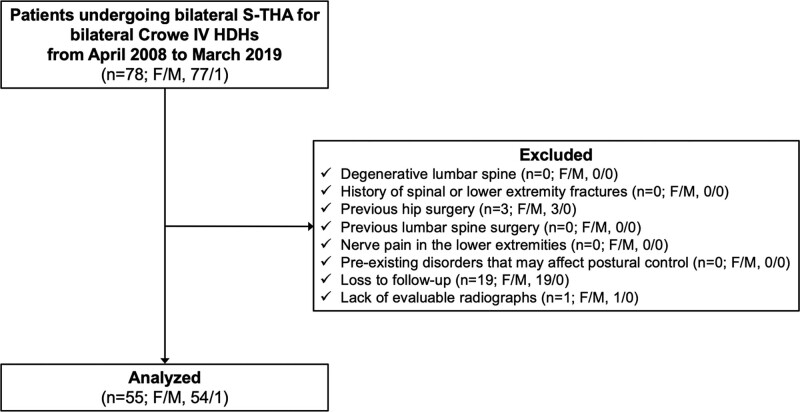
Flow diagram of patient recruitment. F/M, female/male.

We enrolled patients with bilateral Crowe type IV HDHs who required adequate reconstruction with restoration of the anatomical hip center.^[12]^ All patients had undergone bilateral S-THA between April 2008 and Mar 2019. The exclusion criteria were as follows: significant degenerative appearance of the lumbar spine (n = 0); history of spinal or lower extremity fractures (n = 0), previous hip surgery (n = 3), previous lumbar spine surgery (n = 0); nerve pain in the lower extremities (n = 0); preexisting disorders that may affect postural control (e.g., Parkinson disease) (n = 0); loss to follow-up (n = 19); and lack of evaluable radiographs (n = 1). A flow diagram of patient recruitment is shown in Figure 2.

### 2.2. Radiographic study

**Figure 3:**
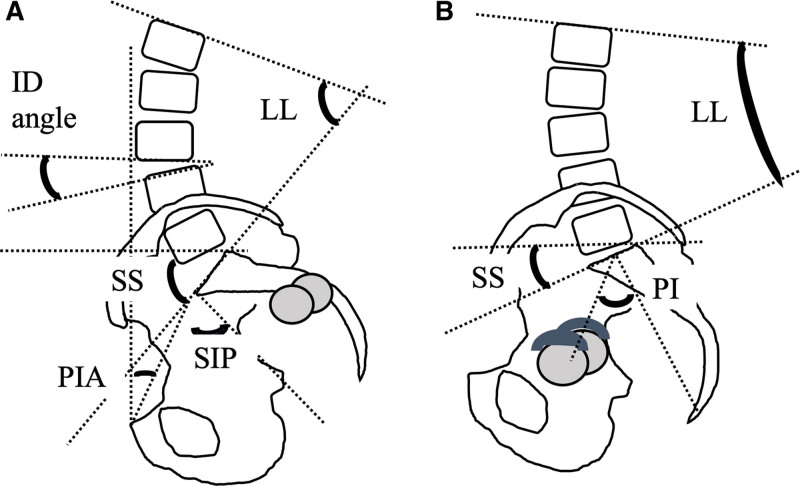
. Illustration of SSPA with Crowe type IV^[4]^ in a highly dislocated hip pre- and post-S-THA. (A) A highly dislocated hip pre-S-THA. The parameters illustrated include LL (L1-S1), SS, ID angle, SIP, and PIA.^[10, 13]^ (B) Illustration of highly dislocated hips post-S-THA. The parameters illustrated include pelvic incidence (PI). In patients with HDHs, the PI can be measured after S-THA because the hip axis is restored to the true acetabulum. HDH, highly dislocated hip; ID angle, intervertebral disk angle; LL, lumbar lordosis; PIA, pelvic inclination angle; SS, sacral slope; SIP, sacral incidence to the pubis; SSPA, sagittal spinopelvic alignment; S-THA, subtrochanteric shortening total hip arthroplasty.

We evaluated the lateral radiographs of the whole spine or lumbar spine and pelvis in a standing relaxed neutral position^[3]^ before and at a minimum of 1 year after S-THA. The SSPA was examined, including the LL, SS, and segmental intervertebral disc angle (ID angle: L1/2-L5/S), before and after S-THA (Fig. 3A and B). ID angles were positive for forward open angles and negative for backward open angles.

In addition, we also investigated the sacral incidence of the pubis, pelvic inclination angle, postoperative PI, and preoperative (predicted) PI, as reported by Imai et al^[13]^ and Takahashi et al^[10]^ (Fig. 3A and B). Sacral incidence of the pubis is the pubic incidence angle, the value of the angle between the line perpendicular to the superior plate of the anterior margin of the first sacral vertebra and the line connecting this point to the superior margin of the pubic symphysis. Pelvic inclination angle is the pelvic tilt angle, the value of the angle between the vertical line and the line connecting the anterior margin of the sacral plate to the superior margin of the pubic symphysis.^[10]^ These parameters have the following geometric relationship: SIP= PIA + SS. Predicted PI was calculated using the following equation: predicted PI=−992+0.95×SIP (R=0.9596, p<0.0001).^[10]^

### 2.3. Statistical analysis

Continuous variables are reported as the mean ± standard deviation (SD) and were compared using the paired *t* test. Pearson correlation coefficient was used to assess the relationships between the parameters.

SPSS (IBM SPSS Statistics for Windows, Version 24.0, IBM Corp., Armonk, NY, USA) was used for the analyses, and a *P* value <.05 was considered statistically significant. A post hoc power analysis for a paired *t* test (setting of α = 0.05 and 2 tails) was performed based on LL and SS, using G* Power 3.1.9.6 (Heinrich-Heine-Universität Düsseldorf, Düsseldorf, Germany). A post hoc power threshold of 80% was used.

## 3. Results

### 3.1. Intraclass correlation coefficient test

Moderate to high intra-observer agreement (0.76–0.92) and inter-observer agreement (0.78–0.96) were observed in all measurements.

### 3.2. Patients demographics

The patient demographics are summarized in Table 1. A total of 54 female patients and 1 male patient (mean age, 63.1 ± 6.9 years) were included in the analysis. The follow-up duration ranged between 12 and 219 months, with a mean duration of 67.9 ± 5.7 months.

**Table 1:** Patient characteristics.

	(n = 55)
Age, yr	63.1 ± 6.9
Sex, male/female	1/54
Follow-up duration, months (range)	67.9 ± 5.7 (12–219)
Preoperative LL (°)	62.3 ± 16.7
Preoperative SS (°)	50.0 ± 12.1
Preoperative (predicted) PI (°)	53.6 ± 10.9
Preoperative L1/2 ID angle (°)	7.1 ± 4.9
Preoperative L2/3 ID angle (°)	10.5 ± 4.4
Preoperative L3/4 ID angle (°)	11.9 ± 4.6
Preoperative L4/5 ID angle (°)	11.2 ± 7.1
Preoperative L5/S1 ID angle (°)	17.4 ± 6.5

ID = intervertebral disc, LL = lumbar lordosis, PI = pelvic incidence, SS = sacral slope.

### 3.3. Comparison test

The mean SSPA values before and after S-THA are summarized in Table 2. Statistically significant differences were depicted between the pre- and post-S-THA measurements of LL (62.3° ± 16.7° vs 49.8° ± 17.0°, *P* < .001), SS (50.0° ± 12.1° vs 39.0° ± 11.1°, *P* < .001), PI (53.6° ± 10.9° vs 48.9° ± 9.6°, *P* < .001), and all ID angles (*P* < .001, for all).

**Table 2:** Comparison of preoperative and postoperative SSPA.

	Preoperative (n = 55)	Postoperative (n = 55)	*P* value
LL (°)	62.3 ± 16.7	49.8 ± 17.0	<.001
SS (°)	50.0 ± 12.1	39.0 ± 11.1	<.001
PI (°)	53.6 ± 10.9	48.9 ± 9.6	<.001
L1/2 ID angle (°)	7.1 ± 4.9	5.6 ± 3.4	<.001
L2/3 ID angle (°)	10.5 ± 4.4	8.4 ± 2.9	<.001
L3/4 ID angle (°)	11.9 ± 4.6	9.1 ± 3.5	<.001
L4/5 ID angle (°)	11.2 ± 7.1	8.9 ± 4.8	<.001
L5/S1 ID angle (°)	17.4 ± 6.5	12.6 ± 5.9	<.001

ID = intervertebral disc angle, LL = lumbar lordosis, PI = pelvic incidence, SS = sacral slope, SSPA = sagittal spinopelvic alignment.

### 3.4. Pearson correlation test

Pearson correlation test results are summarized in Table 3. The correlation coefficients between preoperative LL and SS, postoperative LL and PI, and preoperative (predicted) PI were 0.82, 0.38, and 0.46, respectively (*P* < .001, for all).

**Table 3:** Relationship among the parameters.

	Preoperative LL	Postoperative LL	Preoperative SS	Postoperative SS	Preoperative PI
Preoperative LL	–	–	–	–	–
Postoperative LL	0.82**	–	–	–	–
Preoperative SS	0.78**	0.67**	–	–	–
Postoperative SS	0.31*	0.41**	0.31**	–	–
Preoperative PI	0.42**	0.46**	0.51**	0.26*	–
Postoperative PI	0.41**	0.38**	0.47**	0.22	0.88**

LL = lumbar lordosis, PI = pelvic incidence, SS = sacral slope.

* *P* < .05.

** *P* < .01.

### 3.5. Post hoc power analysis

post hoc power of 96.9% at LL and 99.8% at SS was provided.

## 4. Discussion

In this study, we performed anatomic hip reconstruction with a functional true acetabulum using S-THA for Crowe type IV HDHs and observed the pre- and post-operative SSPA. Our study had 2 main findings in patients with HDHs, as follows: LL (62.3 ± 16.7) and SS (50.0 ± 12.1) were considered high. However, postoperative PI (48.9 ± 9.6) and predicted PI (53.6 ± 10.9) were considered normal as the mean LL, SS, and PI in healthy women aged over 60 years in Japan were reported to be 52 ± 11, 38 ± 8, and 54 ± 10, respectively^[8,14,15]^; post-operative LL, SS, and all lumbar ID angles were significantly improved after S-THA due to restoration of the original hip axis. Moreover, a significant correlation was reported between post-operative LL and post-operative PI, as well as between post-operative LL and predicted PI.

### 4.1. Characteristics of SSPA in patients with HDHs

SSPA is characterized by pelvic hyperanteversion and lumbar hyperlordosis, as reported by Matsuyama et al^[5]^ In addition, there was a strong correlation between preoperative LL and SS (*R* = 0.83, *P* < .01). PI has been reported to increases during childhood, when the spine adapts to bipedal walking, and stabilizes after adulthood.^[16]^ Of note, Hayama et al^[17]^ found that LL and PI were significantly increased in Japanese macaques trained to walk on both legs as infants.

In addition, several authors reported that higher PI was significantly correlated with higher LL and SS.^[8,9,18]^ Therefore, we investigated the possibility that patients with HDHs with large LL and SS may also have a developmentally large PI. However, the PI after S-THA with hip reconstruction on the true acetabulum and the predicted PI without the hip axis were higher in the HDH group. In other words, it can be emphasized that the cause of pelvic hyperanteversion and lumbar hyperlordosis in patients with HDHs is not due to the original PI. Instead, it is a compensatory mechanism to maintain posture for abnormal pelvic morphology, wherein the center of the femoral head was shifted posterosuperiorly due to complete hip dislocation.

### 4.2. SSPA after S-THA in patients with HDHs

S-THA in patients is a procedure that reestablishes the true acetabulum, alters the biomechanics of the lumbar pelvis, and re-creates the relationship between the hip and spine, theoretically allowing for an adaptive reduction in pelvic hyperanteversion and lumbar hyperlordosis (Fig. 1). In this study, the restoration of the original hip axis after S-THA significantly improved the post-operative LL, SS, and ID angles at all lumbar levels. Thus, the shape of the movable disc may contribute to lumbar hyperlordosis.

In contrast, 2 similar prior studies on improving the hip axis for HDHs reported no significant improvement in SSPA.^[6,7]^ They concluded that even though the hip axis was restored, the spine was stiff, with the change being irreversible after many years of HDHs, and could not adjust to the change in the hip center, which did not significantly change the anatomy.^[6,7]^ However, a small number of cases were reported in these studies. Caglar et al reported 27 cases, and Can et al reported on 20 cases. Can et al^[6,7]^ reported that 16 of 20 cases were unilateral dislocations and included a high dislocation (Crowe type III), which was not a complete dislocation. Therefore, the comparison may not be strictly accurate. Caglar et al^[6,7]^ documented that in the same participants as in this study, with all cases operated on being bilateral Crowe IV, the LL changed by an average of approximately 5° post-operatively (*P* = .084). Additionally, they presented a case in which LL was improved by 17° post-operatively. As they have pointed out, this may be owing to the sample size, and if the number of cases increases, it is possible that significant differences will emerge, yielding results similar to those presented in this study.

In addition, the true acetabulum in patients with HDHs may be atrophic, and the cup may not always be placed in the true acetabulum. In such cases, the hip axis may not be reconstructed, which may affect the resultant improvement in the SSPA. In this study, the actual post-operative PI was 48.9 ± 9.6 and predicted PI was 53.6 ± 10.9, indicating that hip-axis reconstruction was generally achieved. The CCs for the correlations between post-operative LL and post-operative PI and between post-operative LL and preoperative (predicted) PI were 0.38 and 0.46, respectively; predicted PI may be highly correlated with post-operative LL. In other words, improvements in LL and SS can be inferred from the predicted PI. This indicates that predictive PI can be specifically effective in cases of femoral head deformity or dislocation where the hip axis is difficult to confirm.^[10]^

## 5. Limitations

First, this was a retrospective study, which was limited by a small sample size and high variability in follow-up time. However, considering the rarity of S-THA in bilateral Crowe type IV hips, this study, which evaluated a total of 55 cases of this rare disease, represents the one with the largest sample size to date. In addition, the post hoc power of the included parameters was considered to be sufficient, as shown in the post hoc power analysis. Therefore, this research endeavor could significantly contribute to improving our insights of the “hip-spine syndrome.” To more accurately observe radiographic changes in the anatomical alignment, a prospective study with timed post-operative surveys would be needed to accurately observe radiographic changes in anatomic alignment. Second, this study did not assess clinical findings because the main focus was on imaging findings. The relationship between low back pain and pre- and post-operative spinal alignment is also an interesting topic for future studies. Third, preoperative and post-operative mobility assessments of the lumbar spine were not performed. Notably, it should be considered that patients with preserved preoperative ID angle mobility may experience greater preoperative and post-operative LL improvements. No reports have examined spinal mobility (stiffness) using dynamic lumbar spine radiography and improvements in SSPA before and after surgery, which could be explored in future studies. Finally, PI was calculated using a predictive formula. However, despite its previously reported high accuracy,^[9]^ this formula is based on normal cases, and whether it can be applied to patients with HDHs remains unclear. In addition, a THA cup may not always be accurately placed in the true acetabulum. Therefore, PI based on the post-operative THA cup may not necessarily reflect the PI in patients with HDHs. A 3-dimensional (3D) pelvic model with computed tomography data may be better for accurate PI measurements.^[19]^

## 6. Conclusion

In patients with HDHs, despite high LL and SS values, the preoperative and post-operative PI values were within the normal range. These findings suggest that in patients with HDHs and preserved spinal flexibility, restoration of the pelvic morphology through S-THA could be a promising approach for improving SSPA.

## Author contributions

**Conceptualization:** Tadatsugu Morimoto.

**Data curation:** Tadatsugu Morimoto, Takaomi Kobayashi, Tomohito Yoshihara.

**Formal analysis:** Takaomi Kobayashi, Tomohito Yoshihara, Hirohito Hirata.

**Investigation:** Tadatsugu Morimoto, Tomohito Yoshihara, Yu Toda.

**Methodology:** Tadatsugu Morimoto, Masatsugu Tsukamoto, Yu Toda.

**Supervision:** Hirohito Hirata, Masaaki Mawatari.

**Validation:** Takaomi Kobayashi, Masatsugu Tsukamoto, Yu Toda.

**Visualization:** Masatsugu Tsukamoto.

**Writing – original draft:** Tadatsugu Morimoto, Takaomi Kobayashi.

**Writing – review & editing:** Hirohito Hirata, Masaaki Mawatari.
